# Systolic spike on transcranial Doppler ultrasound in brain death determination: a matter of numbers

**DOI:** 10.1590/0100-3984.2022.0125

**Published:** 2023

**Authors:** Nicola Morelli, Davide Colombi, Marco Spallazzi, Eugenia Rota, Emanuele Michieletti

**Affiliations:** 1 Neurology Unit, Guglielmo da Saliceto Hospital, Piacenza, Italy; 2 Radiology Unit, Guglielmo da Saliceto Hospital, Piacenza, Italy; 3 Neurology Unit, Hospital of Parma, Parma, Italy; 4 Neurology Unit, San Giacomo Hospital, Novi Ligure, Alessandria, Italy

*Dear Editor,*

We read the review by Corrêa et al.^(1)^ with interest: it is a precise overview of the role imaging plays in the determination of brain death prior to definitive clinical examination. Furthermore, it reviews several methodologies to identify cerebral circulatory arrest. Noteworthy are the data on transcranial Doppler ultrasound, a noninvasive technique which requires no contrast media, as well as being reproducible, inexpensive, and user-friendly, even at the bedside. However, we find the definition of “systolic spike” misleading as it is reported as “short (< 100 cm/s) spikes in the early systolic phase with no flow in the remaining cardiac cycle”. Our considerations are as follows.

Characteristic changes are involved in the development of cerebral circulatory arrest involving the wave form velocity of the basal cerebral arteries^(2)^. Different steps can be identified.

1. *A rise in intracranial pressure*. When the intracranial pressure (ICP) is normal, there is a single-phase flow in systole and diastole toward the brain in the spectra. When ICP increases, there is a reduction in end-diastolic velocity (EDV). Once the ICP reaches diastolic blood pressure, EDV becomes zero. When the ICP is above diastolic blood pressure, the brain receives blood only in systole, during which the systolic flow observed on a sonogram is known as the “systolic peak”. Because the cerebral perfusion pressure is still higher than the ICP, there is still a clear forward flow.

2. *Oscillating flow*. ICP increases continuously, and systolic peak duration is even shorter, thereafter, the diastolic flow is reversed. When the spectra is characterized by systolic forward and diastolic backward flows, the sonogram is said to show “oscillating flow” or a “to-and-fro” pattern. That means that the forward net current is zero, the ICP has exceeded the cerebral perfusion pressure, and the cerebral circulation has stopped.

3. *Systolic spikes*. As the ICP continues to increase, approaching the systolic blood pressure, the diastolic reverse current disappears, and isolated “systolic spikes” appear in the spectra. Systolic spikes must present a systolic peak of < 50 cm/s, rather than 100 cm/s, and a duration of < 200 ms^(3)^, rather than 100 ms.

4. *Lack of signal*. With a very high ICP, the systolic spike amplitudes gradually decrease and in complete cessation of blood flow the signal cannot be observed. The adoption of this pattern in the diagnosis of cerebral circulatory arrest is controversial, because the lack of acoustic signal may be secondary to an inappropriate (overly thick) transtemporal window. However, identification of the flow is a must prior to the flow arrest condition^(4)^.

In conclusion, the specific spectra observed in cerebral circulatory arrest are oscillating flow, systolic spike, and signal absence on the sonogram. In the end, the definition of systolic spike is a matter of numbers (milliseconds).

**Figure 1:**
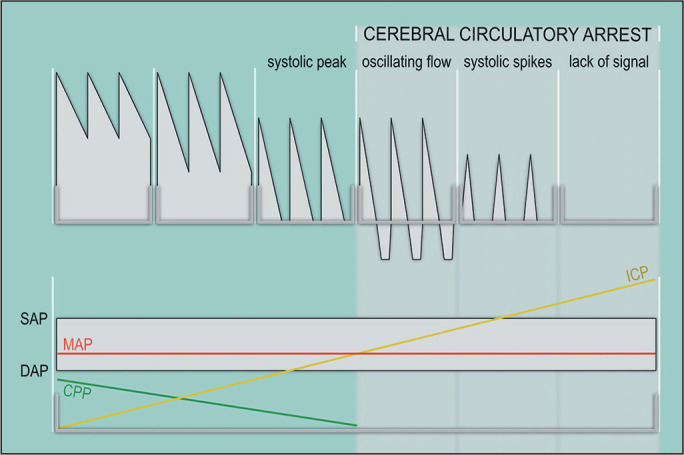
Doppler spectral wave forms from a normal recording to the complete extinction of flow signals due to increasing intracranial pressure. CPP, cerebral perfusion pressure; MAP, mean arterial pressure; SAP, systolic arterial pressure; DAP, diastolic arterial pressure.
