# Supplementary material for: Systolic spike on transcranial Doppler ultrasound in brain death determination: a matter of numbers
Source: Radiol Bras. 2023 Jan-Feb;56(1):50–1. doi: 10.1590/0100-3984.2022.0125 (PMC10013189; doi:10.1590/0100-3984.2022.0125)
Supplement: Related texts

# Reply

**Authors:** Diogo Goulart Corrêa, Simone Rachid de Souza, Paulo Glukhas Cassar Nunes, Antonio Carlos Coutinho Jr., Luiz Celso Hygino da Cruz Jr.

**Affiliations:** 1 Departamento de Radiologia, Clínica de Diagnóstico por Imagem (CDPI)/Dasa, Rio de Janeiro, RJ, Brasil; 2 Departamento de Radiologia, Universidade Federal Fluminense (UFF), Niterói, RJ, Brasil; 3 Departamento de Patologia, Universidade Federal do Rio de Janeiro (UFRJ), Rio de Janeiro, RJ, Brasil; 4 Departamento de Radiologia, Fátima Digittal, Casa de Saúde Nossa Senhora de Fátima, Nova Iguaçu, RJ, Brasil

*Dear Editor,*

We thank Morelli et al. for their interest in our paper on the role of neuroimaging in the determination of brain death, as well as for their comments on the transcranial Doppler ultrasound features of brain death, since this allows a more in-depth debate on the topic. After carefully reading the letter, we understand that the authors concerns are based on the numeric values of the systolic spike pattern, considering the peak velocity at the beginning of systole as well as the duration of this peak. The aim of our article was to review the key neuroimaging features of brain death, with emphasis on computed tomography and magnetic resonance imaging. Because these methods are not considered as ancillary tests in the determination of brain death, they are often disregarded in the assessment of such. Therefore, we did not delve into the technical details and controversies of the other imaging methods.

However, we included all of the neuroimaging examinations considered as ancillary tests for brain death determination in Brazilian Federal Council of Medicine Resolution 2.173, including transcranial Doppler ultrasound. According to the 2014 “Latin American consensus on the use of transcranial Doppler in the diagnosis of brain death”^(1)^, systolic spikes are “very short sonograms with a duration of less than 200 ms, appearing at the beginning of the systolic phase without a flow signal in the remainder of the cycle, and a systolic velocity ≤ 100 cm/s”^(1)^, as we considered in the article. Hassler et al.^(2)^ also described the systolic spike pattern as being characterized by a sharp narrow peak at the beginning of systole, with a maximum flow velocity of up to 100 cm/s. Nevertheless, we recognize that other studies, including the “Brazilian guidelines for the application of transcranial ultrasound as a diagnostic test for the confirmation of brain death”^(3)^, which was published in 2012, define short systolic peaks as those with a peak velocity of less than 50 cm/s^(3,4)^.

In conclusion, we agree with Morelli et al. that the definition of systolic spike on transcranial Doppler ultrasound in brain death is a matter of numbers. Systolic spikes appear at the beginning of the systolic phase without a flow signal in the remainder of the cycle, with a duration of less than 200 ms and a low systolic peak velocity. Some authors define low systolic peak velocity as less than 100 cm/s, whereas others define it as less than 50 cm/s.

## References

[1] Consensus Group on Transcranial Doppler in Diagnosis of Brain
Death (2014). Latin American consensus on the use of transcranial Doppler
in the diagnosis of brain death. Rev Bras Ter Intensiva.

[2] Hassler W, Steinmetz H, Pirschel J (1989). Transcranial Doppler study of intracranial circulatory
arrest. J Neurosurg.

[3] Lange MC, Zétola VHF, Miranda-Alves M (2012). Brazilian guidelines for the application of transcranial
ultrasound as a diagnostic test for the confirmation of brain
death. Arq Neuropsiquiatr.

[4] Escudero D, Otero J, Quindós B (2015). Transcranial Doppler ultrasound in the diagnosis of brain
death. Is it useful or does it delay the diagnosis? Med Intensiva.
